# Splenomegaly in a Patient with a History of Pernicious Anemia; the Potential Therapeutic Effects of B12 Therapy

**DOI:** 10.1155/2022/2854520

**Published:** 2022-04-11

**Authors:** Alexis Lordi, Nida Ansari, Michael Maroules, Anusha Manjegowda

**Affiliations:** ^1^St. Joseph's University Medical Center, 703 Main St., Paterson, NJ 07503, USA; ^2^St. George's University School of Medicine, True Blue, Grenada; ^3^Rowan University School of Osteopathic Medicine, Stratford, New Jersey, NJ 08084, USA; ^4^Department of Hematology and Oncology, St. Joseph's University Medical Center, 703 Main St., Paterson, NJ 07503, USA

## Abstract

Splenomegaly is manifested by a variety of etiologies, one of which is macrocytic anemia. Macrocytic anemia has multiple causes in itself that include; folate (Vitamin B9) and Cobalamin (vitamin B12) deficiencies. In this case report, we present a patient with a history of pancytopenia, macrocytic anemia and vitamin B12 deficiency, who underwent a splenectomy. The differential diagnoses for the cause of the patient's splenomegaly included: lymphoma, infiltrative disease, and idiopathic splenomegaly. The pathology report from the splenectomy did not reveal any evidence of lymphoma or infiltrative disease, however, it did mention vascular congestion of the spleen. In theory, vascular congestion, due to extramedullary hematopoiesis in the spleen or sequestration of blood cell lineages, could lead to pancytopenia. In prior visits to the hospital this patient was diagnosed with: splenomegaly, and macrocytic anemia due to pernicious anemia. A splenectomy puts one at increased risk for infection by encapsulated organisms, and is to be avoided if possible. There are few case reports and studies that show vitamin B12 therapy can potentially cause a reversal in the splenomegaly as well as a reversal in the pancytopenia and macrocytic anemia. We hope to show that the least invasive treatment for vitamin B12, vitamin therapy, can be of use and effective.

## 1. Introduction

Splenomegaly due to exacerbated forms of normal splenic function is common in conditions such as infection, autoimmune disease, liver disease, leukemia, and hemolytic anemia. The spleen has multiple functions; it is involved with hematopoiesis, immunity, and filtering old, malformed, or damaged red blood cells [1]. An increase in red blood cell surface area, as in the case of macrocytic anemia, causes congestion of the splenic vasculature, potentiating pancytopenia. As the spleen works to overcome the congestion, hypertrophy of the organ may ensue resulting in splenomegaly [2]. The patient described in this case report has a past medical history of vitamin B12 deficiency but was non-compliant with treatment, developed progressively worsening splenomegaly, as well as pancytopenia. Initially, it was thought that this patient could have splenomegaly due to infiltrative disease or lymphoma, however, the pathology report was not consistent with either of the differentials and the diagnosis came to a standstill. This case report would like to highlight splenomegaly as one of the manifestations of Vitamin B12 deficiency and that treatment of vitamin B12 deficiency could potentially lead to splenic size regression and reversal of pancytopenia. It has been noted in previous literature, that a patient who had marked splenomegaly and pernicious anemia responded well to vitamin B12 therapy and in three months of vitamin therapy. The spleen was reduced by 2 cm and the pernicious anemia resolved [3]. However, there have not been many presented cases that show the association of splenomegaly and pernicious anemia. We aim to show that with vitamin B12 supplementation, a decrease in the degree of splenomegaly could prevent further, more invasive interventions such as splenectomies in these patients.

## 2. Case

A 48-year-old male with a past medical history of small bowel obstruction followed by exploratory laparotomy, pancytopenia, hepatosplenomegaly, pernicious anemia presented to ED with a chief complaint of left upper quadrant abdominal pain. He reported left upper quadrant pain, decreased appetite, and shortness of breath for a duration of about five to six days. He reported that the pain was exacerbated by deep breaths and that he was unable to walk more than a few feet without feeling short of breath. He additionally reported that his urine had turned dark orange. A urinalysis was done to rule out hematuria or myoglobinuria, which resulted negative for both. The patient reported early satiety and decreased appetite, along with decreased water intake which could be the reasoning for the dark color change of the patient's urine. He stated that he was “play-fighting” with his son and got punched in the stomach 2 weeks prior, but felt fine afterwards. During a previous hospital admission for small bowel obstruction, he was noted to have splenomegaly. He denied any similar presentation in any family members. He denied any allergies to food, medications, latex, dyes. He was not taking any home medications, OTC medications or herbal supplements at the time of presentation. He denied recent alcohol use, although he stated that he used to drink a 6 pack of beer a month. He denied substance use or tobacco use.

**Figure 1:**
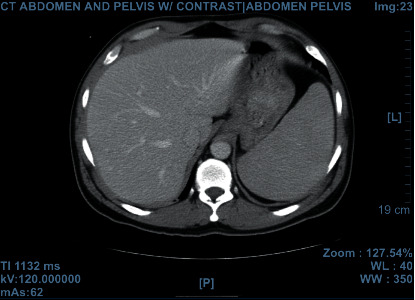
CT scan of abdomen and pelvis with contrast. Diagnosis of splenomegaly, 20 cm. Inferior peri-splenic hemorrhage, three grade laceration.

Upon the initial presentation in the ED, he was in mild distress from the abdominal pain. His vitals were stable. Physical exam revealed mild scleral icterus and sublingual jaundice. His abdominal exam showed a soft, non-distended abdomen, tenderness to palpation in LUQ, dullness to percussion in LUQ, spleen was palpable up to 3-4 inches below the rib cage, no hepatomegaly, no rebound, rigidity or guarding was appreciated. The remainder of the physical exam was unremarkable. Abdominal CT (Figure 1) showed increased splenomegaly from 17 cm previously to 20 cm currently, with inferior peri-splenic hemorrhage and the linear plane of hypoattenuation consistent with grade 3 laceration.

On this current admission, his lab results showed macrocytic anemia with hemoglobin of 6.9 g/dL, hematocrit of 21.7% and MCV of 101.4 fL. Patient was found to have a WBC of 2.1 × 10^3/mm3 indicating neutropenia but denied fever or recent sick contacts and thrombocytopenia of PLT: 125 K/mm3. His anemia panel showed no evidence of iron deficiency, B12 level was 137 pg/mL, folate level was 4.9 ng/mL. Patient also had lab findings of coombs negative hemolytic anemia with LDH of 560 unit/L, reticulocytosis of 17%, haptoglobin of <10, total bilirubin of 5 mg/dL secondary to vitamin B12 deficiency. The initial MMA was elevated at 385 and homocysteine was 11.8 about 2 years prior to this presentation. Patient had elevated levels of intrinsic factor antibodies and was diagnosed with pernicious anemia. He had been non-compliant with taking B12 supplements. On further investigation, it was found that in previous admissions he had an extensive hematologic workup including JAK testing, BCR-ABL testing, peripheral blood flow cytometry, and bone marrow aspirate and biopsy which was normal. On this admission, hemolytic workup resulted as a urobilinogen within normal limits, normal ALT, but an elevated LDH and positive reticulocytosis. A blood smear was not done at the time of hemolytic workup, but the splenic laceration could be the cause of a mixed acute hemolytic picture. Previous lab work would suggest that his hemoglobin count is consistently low; ranging from 9.4 to 11.7 since 2019.

Patient underwent a splenectomy for the grade three splenic laceration. Pathology showed markedly enlarged spleen weighing 1260 grams with marked vascular congestion and measuring 22 × 16 × 7.5 cm. There was no evidence of myeloid or lymphoid proliferative disease. It was concluded that the case of splenomegaly was caused by B12 deficiency secondary to pernicious anemia and non-compliance with vitamin supplementation. The patient was restarted on vitamin B12 intramuscular injections.

## 3. Discussion

Vitamin B12, also known as Cobalamin, deficiency classically causes macrocytic anemia. It has been hypothesized that the increase in size in red blood cells from macrocytosis can lead to congestion of the red pulp in the spleen and sequestration of other blood cell lineages [2]. Sequestration of the oversized red blood cells in the spleen over time in this patient leads to splenomegaly [2].

Vitamin B12 deficiency can be secondary to dietary restrictions such as a strict vegetarian diet, or autoimmune pernicious anemia. Once cobalamin is released from parietal cells, Intrinsic Factor forms a complex with vitamin B_12_ in the duodenum for absorption in the ileum to occur [4]. In a patient with pernicious anemia, autoantibodies are formed to prevent the release of Intrinsic Factor, therefore decreasing the amount of absorbed cobalamin [4]. When there is a decrease in the amount of available vitamin B12 there is a decrease in DNA synthesis, multilinear blood cell proliferation, and cellular metabolism. This results in ineffective erythropoiesis, leading to extramedullary hematopoiesis as a compensatory mechanism, manifesting as hepatosplenomegaly. [5] Extramedullary hematopoiesis typically occurs in the red pulp of the spleen. Clinical Manifestations of vitamin B12 deficiency include; shortness of breath, fatigue, pallor, loss of memory, poor balance, reduced sensation of touch, and tingling feet. [2]

Gedik et al. claim that out of 137 participants with pancytopenia, vitamin B12 deficiency was found to be the etiology in 24 of the participants [6]. It was the most frequent cause of pancytopenia. Halfdanarson et al. stated that severe megaloblastic anemia can be mistaken for leukemia which was also part of the workup for this patient but no significant pathology was found [3]. The initial presentation of vitamin B12 deficiency including pancytopenia, splenomegaly and leukoerythroblastosis is a common concern when considering a diagnosis of leukemia [3]. Our patient also had a blood smear that demonstrated leukoerythroblastosis, which is also described in the setting of infiltrative disease. In the Halfdanarson et al. article, a bone marrow examination of Cobalamin deficiency showed marked hypercellularity, with an increase in erythroid precursors, megaloblastic maturation, and a decreased amount of granulocytes. The megakaryocytes were normal in number but contained large nuclei. [3]. This case report reminds us to look into nutritional aspects when coming across a patient with splenomegaly and the pathology report does not show the clinical finding that was expected.

Pruthi, Rajiv K. et al. describe a patient who had marked splenomegaly, similar to this patient, and after three months of vitamin B12 therapy the spleen had decreased in size and the anemia had resolved [7]. A case report by Behera, Vineet, et al., describes a patient with megaloblastic anemia and a spleen palpable 13 cm below the left costal margin was treated with parenteral vitamin B12 1,000 mcg twice a week intramuscularly. Follow up at 1, 3 and 6 months showed sizeable improvement in his condition. As well as, serial abdominal ultrasounds showed gradual decreases in splenic size. A regression of 2 cm after 6 months was reported [1]. Our patient began to show signs of recovery of his blood counts within 3-4 weeks of parenteral vitamin B12 supplementation. He also had progressively worsening splenomegaly and pancytopenia in the setting of non-compliance with vitamin B12 therapy indicating the role of vitamin B12 deficiency in the development of splenomegaly. It is important to rule out B12 deficiency in the right clinical setting in all cases of splenomegaly to make sure that the patient is getting adequate B12 supplementation as it is the least invasive tool to reduce the size of the organ and in some cases, as seen above has been proven efficacious [7].

## 4. Conclusion

Splenomegaly is a rare manifestation of pernicious anemia. One of the major mechanisms is believed to be due to congestion in the red pulp of the spleen with macrocytic red blood cells which was seen in our patient. There have been few cases reported of pernicious anemia with concurrent splenomegaly reported as of 2014. It can be hypothesized that this patient's untreated B12 deficiency allowed for pancytopenia to persist secondarily from the vascular congestion causing splenomegaly. Hypothetically if this patient did not have a splenic laceration leading to a splenectomy, and had he been more adherent with B12 injections, the splenomegaly could have been reduced or at least controlled. Three months later, it was found that his pancytopenia had resolved. Previous literature demonstrates that it is possible to reverse splenomegaly secondary to B12 deficiency. This is something to consider in the management of those who present with B12 deficiency and splenomegaly.
